# Esophageal Foreign Bodies in Pediatric Patients: A Thirteen-Year Retrospective Study

**DOI:** 10.1100/2012/102642

**Published:** 2012-04-19

**Authors:** Beata Rybojad, Grazyna Niedzielska, Artur Niedzielski, Ewa Rudnicka-Drozak, Pawel Rybojad

**Affiliations:** ^1^Department of Anesthesiology and Intensive Care, Children's University Hospital of Lublin, Chodzki Street 2, 20-093 Lublin, Poland; ^2^Department of Pediatric Otolaryngology, Phoniatrics and Audiology, Medical University of Lublin, Chodzki Street 2, 20-093 Lublin, Poland; ^3^Independent Laboratory of Medicine of Disasters, Medical University of Lublin, Chodzki Street 6, 20-093 Lublin, Poland; ^4^Department of Thoracic Surgery, Medical University of Lublin, Jaczewskiego Street 8, 20-954, Poland

## Abstract

We discuss clinical symptoms and radiological findings of variable esophageal foreign bodies as well as therapeutic procedures in Caucasian pediatric patients. A retrospective study of 192 cases of suspected esophageal foreign bodies between 1998 and 2010 was conducted. Data were statistically analyzed by chi-square test. A foreign body was removed from a digestive tract of 163 children aged 6 months to 15 years (mean age 4.9). Most objects were located within cricopharyngeal sphincter. Dysphagia occurred in 43%, followed by vomiting (29%) and drooling (28%). The most common objects were coins. Plain chest X-rays demonstrated aberrations in 132 cases, and in doubtful situations an esophagram test was ordered. In the group of thirty-seven patients whose radiograms were normal, esophagoscopy revealed fifteen more objects, which were eventually successfully removed. No major complications occurred. Esophagram should be a second X-ray examination if an object is not detected in plain chest X-ray. We recommend a rigid esophagoscopy under general anesthesia in doubtful cases as a safe treatment for pediatric patients.

## 1. Introduction

Even though esophageal foreign bodies (EFBs) in children stay an important and difficult medical subject, there are no official statistical data concerning foreign body (FB) ingestion in Poland. In the USA, there are over 100 000 cases of EFBs per year and 1500 of these patients die [1–3]. In 80–90%, an FB passes spontaneously through an upper gastrointestinal (UGI) but sometimes it lodges in the esophagus and needs to be removed to avoid dangerous complications: obstruction or perforation of the UGI, bleeding, ulcerations, or fistulas [4]. Destruction of a thin and fragile esophageal wall by an ingested sharp FB or iatrogenic maneuvers may increase a complication rate from 1% to 35% or may lead to mediastinitis, which is highly lethal. In children, only 40% of FBs pass asymptomatic [3]. There are different methods of removing EFBs. Rigid esophagoscopy is one of them, but in children it requires a general anesthesia. Flexible endoscopes may be used without an anesthesia, but if a sharp FB is suspected, it might be dangerous to pull such an object behind the endoscope because of the risk of perforation. In such cases, classical, rigid endoscopes are used and a sharp object is hidden inside the tube. There are also some other, alternative methods of FB removal. They can be used in cases of extreme emergency when more sophisticated equipment is not available. Some physicians [5] try to remove FBs with the use of Foley's catheter, especially when an object seems to be oval in shape (e.g., coins, small balls). The advantages of this method are simplicity and accessibility. It is also time and money saving because using of the Foley's catheter does not require general anaesthesia and can be performed even shortly after a meal. However, this method is recommended only for removal of oval objects. In our hospital, a rigid esophagoscopy is provided as a golden standard for EFBs removal. It is performed under general anesthesia, after fasting time (approximately 6 hours) if there are no urgent recommendations (e.g., a sharp object, a button battery lodged over 12 hours). Otherwise, an esophagoscopy is done as soon as the operating and anesthetic teams are ready. Although this method is more difficult and requires high experience from the performing doctor, it also enables to avoid tearing of the thin esophageal mucosa by sharp objects which can be drawn inside the esophagoscope lumen [6]. Children constitute the large group of patients with EFBs because of the way they get to know the surrounding world. Teenagers have habits of keeping pen tops or pins in mouths, during their routine daily activities. Toddlers sometimes swallow objects to draw attention to their parents on themselves, especially when a newborn baby appears in their family. An FB swallowed as a self-mutilation method occurs much less frequently among children than among adults [7–10]. Radiological examination is helpful in identification of a kind and location of EFB. Posteroanterior and lateral chest and neck X-ray show radiopaque objects: metal coins, jewellery, and batteries. The presence of the radiolucent foreign bodies in UGI can be detected by esophagram test with contrast (barium). The aim of the study was to present EFB cases treated in Children's University of Lublin within 13 years and to analyze the most frequent clinical symptoms and reliability of diagnostic procedures after FB ingestion.

## 2. Material and Methods

**Figure 1:**
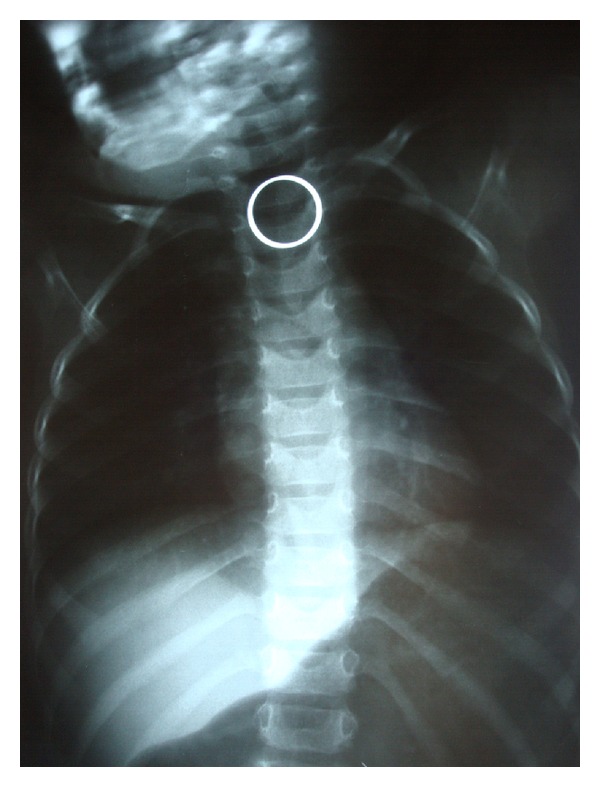
A radiopaque FB (a wedding ring).

 We analyzed medical documentation of 192 patients with the suspicion of an EFB, hospitalized in years 1998–2010 in the Department of Pediatric Otolaryngology, Phoniatrics and Audiology, Medical University of Lublin. Medical history, clinical symptoms, and the radiological examinations constituted a base for qualification of the patient for the endoscopical procedure. Sometimes an EFB was difficult to be diagnosed, and a physician mostly had to rely on the anamnesis from the child and parents. The most common clinical symptoms described by them were drooling, vomiting, dysphagia, neck, throat, or chest pain, and cough. In all our patients, a plain chest and neck X-ray was performed (Figure 1).

**Figure 2:**
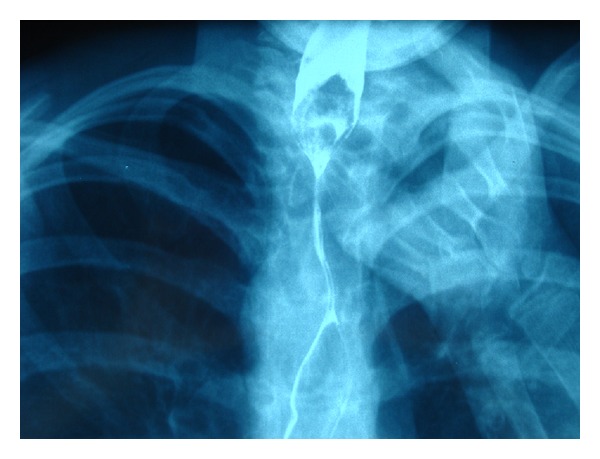
A radiolucent FB (a peach seed) within first esophageal narrowing.

In doubtful cases, a lateral chest and neck profile was X-rayed additionally. If a suspected FB was still invisible, a barium swallow test was done (Figure 2).

**Figure 3:**
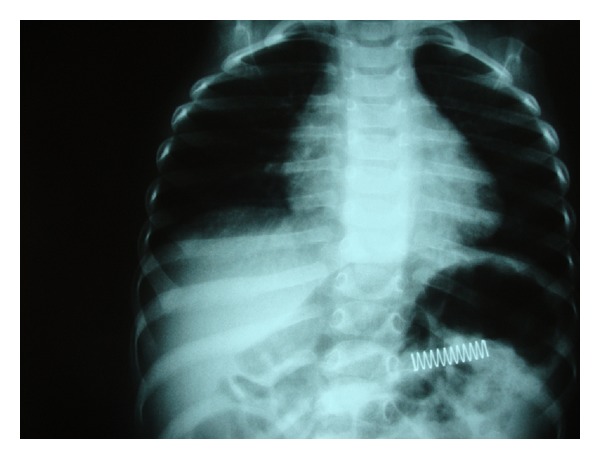
A spring which was later expelled spontaneously.

 Sometimes an abdominal X-ray was performed to locate an FB which already had passed into further parts of GI (Figure 3).

 Esophagoscopy was usually conducted within 8 hours from admitting into hospital (depending on the last meal and the availability of the anesthesiological team). Each patient was given a general anesthesia with intubation and muscle relaxation. After removing an FB (or seeing it passing further into the stomach), a second endoscopic look was taken to control the mucosa of the esophagus. Esophagoscopy was performed in 192 children with an FB suspicion. The largest population was treated in 1998—27 patients, the smallest one in 2010—6 patients. We divided the examined patients' documentation in three age groups: under 1 year old, between 1 and 3, and over 3 years old. An age, gender, kind of the foreign body, clinical symptoms, and radiological findings were analyzed. We also considered location of the FB in gender and age groups. We specified 3 locations depending on anatomical narrowings: first in a cervical esophagus (cricopharyngeal sphincter), second—crossing with the aortic arch, at the bifurcation level and third—esophageal hiatus (gastroesophageal junction) [11, 12]. Relations between clinical symptoms and the location of FBs were noted. In our survey, classification of FBs was conducted by taking into account their origin (organic, inorganic) and the radiological visibility (radiolucent or radiopaque). We also noted groups of most often removed objects: coins, parts of toys, jewellery, and fragments of food. Relations between the location of the foreign body and appearing clinical symptoms were examined. A usefulness of radiological examinations was analyzed.

### 2.1. Statistical Analysis

The results were statistically analyzed with the use of the chi-square test (*χ* ^2^). A *P* value of less than 0.05 was considered significant. The multiway tables were drawn.

## 3. Results

FB was removed from the esophagus in 163 cases (84.9%). In the analyzed period, 128 boys were being hospitalized (67%) at the age from 6 months up to 18 years. Males dominated in each year group. Objects were mainly located in the first narrowing (44%), followed by the second one (23%). Children between 1 and 3 years old constituted 44.6% of population (Table 1).

Coins (54%), fragments of food (19%), parts of toys (7%), and jewellery (4%) were the most often removed objects. In the group of “Other,” we included mainly radiopaque FBs: button batteries, pen tops, screws, paperclips, and so forth.

 Depending on location, different clinical symptoms dominated. Objects impacted in 1st narrowing caused mainly drooling, vomiting, and dysphagia. Significant statistical correlation was stated between the location of a FB and the clinical manifestations. Drooling and vomiting significantly more often have appeared when FBs were located in the 1st narrowing. Pain complaints significantly more often appeared when FBs were located in the 3rd narrowing of esophagus (Table 2).

 Plain chest radiograms confirmed the presence of 132 radiopaque objects (68.8%) except two coins which were detected during esophagoscopies.

**Figure 4:**
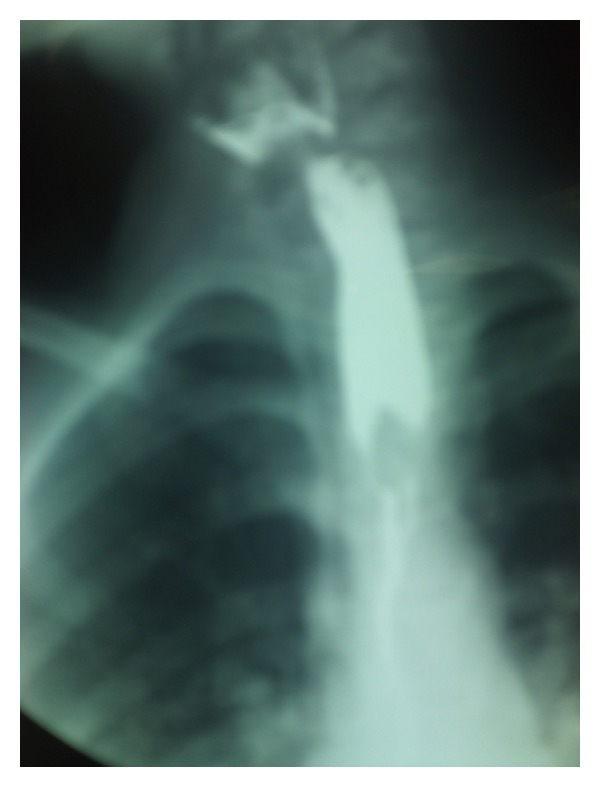
A clove of garlic swallowed as a pinworms treatment.

Additionally done esophagram showed probable locations of 17 radiolucent FBs. If an FB blocked an esophagus, contrast stopped above the object (Figure 4).

Although in 39 cases (20.3%) X-rays were normal, in 17 of them an FB was removed during esophagoscopy. In twenty-six patients (13.5% of examined population), whose plain chest X-rays before the treatment shown FBs (coins visible in 3 variable locations), we did not find any FB during esophagoscopy, and on the radiograms done afterwards, coins were seen in the stomach.

We stated highly significant statistical correlation between the kind of the foreign body and radiological findings (*P* = 0.0001). Inorganic foreign bodies were significantly more often visible on X-rays. Radiograms as asymptomatic were more often described after aspiration of organic foreign bodies. Indirect manifestations, significantly more often appeared in radiological images after aspiration of organic objects. (Table 3).

We noted only 22 esophagoscopies with slight bleeding (11.4% of all cases) and 12 procedures showed erosion of mucosa (6.3%). In three children (1.6%), breathing problems after the procedure occurred. There were no major complications needed surgical interventions.

## 4. Discussion

 As other authors [12–15], we divided an analyzed population in three age groups: infants 1–12 months, children between 1–3 years of age, and children older than 3. In our material, we noted the slight domination of children over 4 years old, oppositely to quoted authors, where children between 1 and 3 years of life dominated. Infants constituted 6.5% of the whole population, and it was similar to other studies [2, 6, 7, 10, 15–17]. Amongst 192 cases of our survey, FBs were stated in 163 patients (84.9%). It is comparable with the quoted authors [3, 7]. Al-Quadach and colleagues [12] were very successful—an FB was removed in 93.8% and only 5.4% pushed down to the stomach. Sometimes an FB visible on an X-ray before the esophagoscopy passed to the stomach during procedure. It happened in 26 cases (13.5%) in studied material. Other authors also mentioned about passing down of the objects [18–20]. The cause of this phenomenon may be explained by propulsive waves, which appear in the UGI during esophagoscope insertion [9].

FBs during endoscopies were most often found within the first narrowing of the esophagus, which is similar to what as other researchers stated [6, 16, 18, 20, 22–24]. Children with the impaired reflex of swallowing, mentally retarded or after operations of congenital defects of the esophagus (atresia with the tracheoesophageal fistula or without the fistula), have predispositions to aspiration of foreign bodies. Kay and Wyllie [2] mention that in USA about 2% of FBs are found in retarded children. In our study, there were 6 retarded patients (3.1%) and in two of these cases coins were removed. Reading [21] described a case of a retarded, deaf, mute, tetraplegic boy, who had not eaten any meals but fluids and an FB removal returned him an appetite. In our study there were three boys with a constricted esophagus after chemical burns and two patients after a plastic surgery of congenital atresia of esophagus with tracheoesophageal fistula. One boy from that group ingested an FB three times within three years and each time needed an esophagoscopy under general anesthesia. Similar cases were described by other authors [6, 9, 10, 12, 20].

 We notified that independently from the location of a FB, most of our patients were males (67%). Conners [22] showed comparable results. This gender disproportion might come from different temperaments of boys and girls and the ways of playing. However, in one publication, the authors [2] noted that girls were the more numerous group.

 Clinical symptoms accompanying EFBs depended mainly on the location, size, and time elapsed from the accident. Directly after ingestion strong cough was observed, sometimes with vomiting. Dysphagia occurred in eighty-three children (43%), which was in correlation with some surveys [20, 22, 23]; however, in other studies lower frequency, 26–37%, was noted [18, 24]. Those authors usually wrote about hypersalivation which occurred in 31–46%. In our survey, it was observed only in 28% (fifty-four patients) [18, 24]. Vomiting in case of logged EFBs is dangerous, because the pressure may cause the rupture of the thin wall of the esophagus. This manifestation clearly diversified centers of individual researchers. We noted this symptom in 56 patients (29.1%). It was not troublesome in the research of Balci and coworkers [18], but Al-Qudah et al. [12] reported it was one of the two most common symptoms.

In our study, pain occurred only in thirty-seven patients (19.2%). It was mostly located in the chest and upper abdomen and it correlated with the location of an FB. In Little's survey [25], a sore throat and chest pain were essential and nagging symptoms. Pain may be the only sign of an FB that was ingested not witnessed [5]. Łasiński reported that in the literature review 7–35% of patients had no ingestion symptoms and finally esophagoscopy proved an impacted object [11]. In our study, there were 5 asymptomatic patients (2.6%) with an FB ingested. Athanassiadi and colleagues [20] noted neither false-negative nor false-positive radiological findings.

Radiological examinations constitute the essential diagnostic method in case of the identification of an FB. A plain chest X-ray in lateral and posteroanterior position (PA), or anteroposterior (AP) in infants, is a basic diagnostic procedure [25]. Objects made of metal are radiopaque (coins, safety pins, batteries, jewellery). Plain chest X-rays showed FBs in 134 cases, additionally made esophagograms allowed for detecting 17 more objects. In thirty-seven children although X-rays were normal, fifteen FBs were removed during esophagoscopy.

In our hospital inorganic objects were most often removed from the esophagus (mostly coins), which is similar to other European countries and the USA [7, 9, 12, 18, 20–22]. Coins were visible in 89 photographs of our patients (53%). In analyzed material, there was one exceptional case of three-year-old who swallowed coins. Diagnostic procedures revealed only 3 visible coins in the esophagus, esophagoscopy enabled removal of two more coins from the esophagus (total number of evacuated coins was 5). In Asian countries, the assortment of FBs differs because of dietetic habits. Some authors report that majority of objects removed from the esophagus in children in the Hong Kong and India was of organic origin: fish and chicken bones [24, 26]. Nayak et al. [26] notified FBs in the esophagus in 48% of survey plain X-rays, and on esophagrams with contrast this value grew up to 53%. Balci and co-workers [18] reported as many as 93% visibilities of FBs (mostly coins).

In our study, esophagrams showed the obstacle in the esophagus of seventeen children. One barium swallow test did not show an FB; nevertheless, a plastic button was removed from the first narrowing of the esophagus. Some objects, especially of smooth shape (balls, coins), spontaneously passed to the stomach in the period between the performed radiogram and esophagoscopy, so Kay and Wyllie [2] as well as our team recommend an X-ray examination right before the procedure to avoid the unnecessary risk for the patient. Ten children were admitted with the suspicion of the sharp FB in the esophagus, but in seven cases the examination excluded the presence of the bone or bones in the upper stretch of UGI (probably passed by to the further parts of the GI).

## 5. Conclusions

 We would like to stress on the importance of good anamnesis concerning the kind of FB, possible personal factors predisposing to FBI (e.g., congenital fistulas, chemical burns in past). Even a normal plain chest and a cervical X-ray followed by an esophagram does not exclude the presence of an FB. Radiological examinations and anamnesis are helpful to diagnose EFBs in children but sometimes they give false positive or negative results. This is the reason we recommend a rigid esophagoscopy under general anesthesia for eventual diagnose and treatment. Despite various alternative methods of FBs removal, a rigid esophagoscopy remains a “golden standard” as a safe and efficient method of removing objects from the esophagus of pediatric patients.

## Figures and Tables

**Table 1:** Location of an FB concerning age and gender.

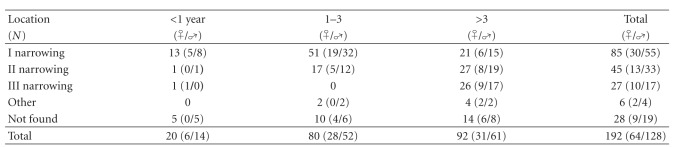

(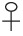 **—**female, 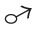 **—**male).

**Table 2:** Most frequent clinical symptoms of esophageal FB, depending on location.

Location	Drooling (*N*)	Vomiting (*N*)	Dysphagia (*N*)	Pain (*N*)
I narrowing	34	34	33	6
II narrowing	12	13	24	9
III narrowing	2	3	8	12
Other	4	3	9	4
Not found	2	3	9	6

*χ* ^2^	13.99	14.68	8.19	23.23

*P*	0.015654	0.00820	0.08473	0.00011

**Table 3:** Visibility of FB depending on its origin (organic/inorganic).

Kind of FB	Plan chest X-ray findings		No findings		Indirect findings (esophagogram)		Total	
	*N*	%	*N*	%	*N*	%	*N*	%
Organic	2	1.5	23	56.1	11	64.7	36	18.7
Inorganic	132	98.5	18	43.9	6	35.3	156	81.3

*χ* ^2^ = 86.21978								
*P* = 0.00001								
